# Teledentistry as an Effective Tool for the Communication Improvement between Dentists and Patients: An Overview

**DOI:** 10.3390/healthcare10081586

**Published:** 2022-08-21

**Authors:** Md Refat Readul Islam, Rafiqul Islam, Sultana Ferdous, Chiharu Watanabe, Monica Yamauti, Mohammad Khursheed Alam, Hidehiko Sano

**Affiliations:** 1 Department of Restorative Dentistry, Graduate School of Dental Medicine, Hokkaido University, Kita 13, Nishi 7, Kita-ku, Sapporo 060-8586, Japan; 2 Department of Restorative Dentistry, Faculty of Dental Medicine, Hokkaido University, Kita 13, Nishi 7, Kita-ku, Sapporo 060-8586, Japan; 3 Department of Neurobiology, Graduate School of Medicine, Hokkaido University, Kita 15, Nishi 7, Kita-ku, Sapporo 060-8638, Japan; 4 Preventive Dentistry Department, College of Dentistry, Jouf University, Sakaka 72345, Saudi Arabia

**Keywords:** teledentistry, healthcare, real-time videoconferencing, digital dentistry, digital health, COVID-19

## Abstract

Teledentistry is an online dental care service that allows patients and dentists to meet in real time, safely, without being at the same location. During the COVID-19 pandemic, real-time videoconferencing has gained popularity in the field of teledentistry, with numerous benefits for both patients and dentists. Online consultations can minimize costs, maximize time, and provide more convenient care options for both patients and dentists. When practicing teledentistry, a dentist must establish a good doctor–patient relationship. Dentists must ensure that the telecommunication solution that they choose meets their clinical requirements and complies with privacy laws. Dentists should provide adequate information to patients about the limitations, advantages, and disadvantages that may occur during online consultation. Dentists must follow guidelines and procedures regarding informed consent, patient details, personal communications, and consultancies’ privacy and confidentiality. The patient should be aware of the limitations of teledentistry, and dentists will provide the best advice possible in the absence of a face-to-face consultation. This article discusses how teledentistry could be an effective tool for dentists and patients.

## 1. Introduction

**Figure 1:**
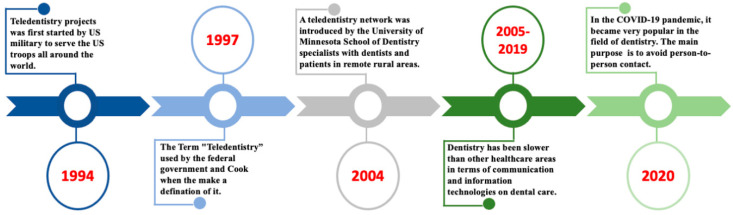
Evaluation timeline of teledentistry.

With the era of technology and telecommunication, the healthcare system is changing rapidly. Several telecommunications systems have been implemented for hospitals and, with the passage of time, a new term for it has emerged, namely “telemedicine” [1]. As a branch of telehealth, telemedicine employs communications networks from one regional area to another to deliver healthcare facilities and medical education, especially to solve problems such as unequal access and a lack of infrastructure and human resources [2]. Teledentistry is a subfield of telehealth, along with telemedicine, that focuses on dentistry, derived from interactive tools, telecommunications, and dentistry combinations [2]. Teledentistry uses information technology to facilitate remote dental care, advice, education, or treatment rather than direct face-to-face communication with patients [3]. The first study on teledentistry was conducted in 1994. It was conducted by the US Army as part of the US Army’s Total Dental Access Project [4]. Cook et al. defined the term teledentistry in 1997, as “the practice of diagnosing and providing treatment advice over a distance using video-conferencing technology” [5] (Figure 1). 

It can be used as a service modality in three primary ways [6]. These include the following: (i) consultations among dentists—for example, a general dentist and a specialist dentist can exchange patient photos and records, followed by a review and treatment planning discussion; (ii) a real-time face-to-face video conference consultation between a general dentist or specialist and a patient or family member in a distant, remote location; (iii) remote patient monitoring, collecting data in real time and transmitting them to the dentist in a remote location for examination and action as required [6]. It also assists in providing patients with basic knowledge about dental healthcare and improving patients’ healthcare facilities [7,8]. This facilitates the efficient exchange of information and knowledge between the patient and the doctor and among different specialists to achieve better treatment planning and outcomes [9]. Most dentists or specialists prefer synchronous or real-time videoconferencing with the patient through teledentistry systems [10]. In the healthcare system, real-time interactive consultations are now integrated [11]. Several studies have indicated that real-time video consultations play an important role in the healthcare setting and, in these cases, they also demonstrate advantages over simple mobile consultations [11,12]. Recent studies showed that video consultations were thought to be useful for general practitioners but perhaps more suitable for particular groups of patients [13]. The demand for online video consultation is likely to increase, but it is essential to improve the technical infrastructure and to select cases [14]. Therefore, real-time interactive video consultation is the most authentic means of communicating with patients and conducting a teledentistry examination. During online consultation, a dentist must establish a doctor–patient relationship with the patient. When establishing relationships between dentist and patient, it is crucial to maintain the proper guidelines or recommendations for online consultation from both sides. In this article, we discuss how teledentistry could be an effective solution for dentists and patients.

## 2. Technologies Used in Teledentistry

Information and communication technology (ICT) is being developed day by day. Teledentistry can help in oral healthcare almost in every corner of the world. It connects the patient, dentist, and specialist from the relevant fields, where exchanges and interactions are established for diagnosis and treatment. “Store-and-forward” and “real-time” are the two main types of interaction. “Store-and-forward” is commonly referred to as pre-recorded media or asynchronous systems, whereas real-time interactions are known as synchronous systems [15].

Clinical information, including clinical images, can be gathered in a virtual storage place in the store-and-forward system, which can be further used as a diagnostic reference and treatment plan [16]. Moreover, it can be a valuable tool in patient counseling, even if the patient is far from the consultation process [17]. Dentists and specialist clinicians can easily exchange patient information, radiographic images, pathological images and laboratory findings, treatment modalities, and other information related to oral and general health status. They may seek help from a different specialist for their opinions and counseling. Usually, low-bandwidth internet is suitable for such kinds of systems. 

In real-time methods, a consultation can take place via video calling or conferencing between a dentist, specialist clinicians, and a patient. They can quickly review clinical and general information, medical history, radiographic images, and laboratory findings for diagnosis. This interactive approach allows real-time images and better understanding from the doctor’s side to the patient’s side [1,17,18,19,20,21].

Remote patient management is a recent addition to this field. In this method, dentists or other clinical experts can obtain detailed information directly from patients’ homes about their health conditions and easily send them to clinical centers. The main advantage of this method is that it reduces healthcare costs.

Moreover, recently, mobile communication devices have gained attention in the field of public health education and promotion, especially cell phones and tablets. 

## 3. Applications of Teledentistry

### 3.1. In Diagnosis

The most significant aspect of teledentistry is its ability to minimize healthcare disparities, improve patients’ access to professional advice, shorten the treatment duration without sacrificing quality, and drastically reduce waiting times [15,22]. With the introduction of teledentistry into oral health services, patients might have easy access to diagnosis and management of their oral health concerns [10]. One of the possible reasons for the delayed diagnosis of oral cancer is the improper diagnosis of oral lesions [23]. In this regard, teledentistry aids in the early detection of malignant lesions, enabling faster measures for the treatment of oral cancer and enhancing the effectiveness and safety of the therapy [24] by facilitating communication between dentists and clinical specialists. In addition, remote diagnosis can be a useful method for detecting oral lesions [4,25,26]. Involving many specialists in the diagnostic process seems to be a promising approach for increasing the precision of remote diagnosis [8,25,27].

### 3.2. In Oral and Maxillofacial Surgery 

Previous research has demonstrated that teledentistry can be an effective technique for the purposes of consultation, assessment of the patient, treatment planning, and follow-up care in maxillofacial surgery [28,29,30,31,32]. Patients’ and dental surgeons’ acceptability regarding teledentistry performed through telephone appointments for different patient categories in maxillofacial surgical practice in comparison with the on-site evaluation was highly appreciated [33]. This study also showed that most of the patients with conditions such as temporomandibular disorder, salivary gland disorders, head and neck cancer, and orthognathic surgery require further assessment owing to the difficulty of carrying out a standard “face-to-face” consultation. Other studies revealed the overall perspectives of both dentists and patients on teledentistry [34,35]. They reported that teledentistry was generally well accepted by patients with trauma, whereas the lowest acceptance was found in patients with temporomandibular joint disorder. Concerning how dentists and clinicians perceive teledentistry, most of the dentists were happy with it. However, some of them expressed concern about its effectiveness because the inability to perform physical examinations can lead to an incomplete diagnosis. 

### 3.3. In Orthodontics

Teledentistry is becoming more popular in orthodontics as a means to consult with and monitor patients without visiting dentists due to technological advancements. Virtual consultation and artificial intelligence (AI)-based treatment monitoring methods with photos or videos are currently the main forms of online communication technology used in orthodontics [36]. Regarding initial orthodontic consultations, teledentistry can be particularly useful in evaluating treatment options and diagnosis plans, measuring calibration and orientation, and assessing functional appliances and removable appliances. Although teledentistry has not completely eradicated the need for in-person clinical care, most of the dentists and patients believe that online consultations or teledentistry are more convenient and cost-effective in orthodontic treatment [37,38]. Minor orthodontic emergencies such as rubber ligature displacement, pain, and cheek irritation can be handled at home via teledentistry, reducing the need for in-person visits to the dental clinic [39]. Nevertheless, many procedures that need direct access to the oral cavity cannot be performed by teledentistry, despite having unique advantages. For instance, an orthodontist may carry out a preliminary examination such as gathering data, installing brackets and bands, bonding and removing them, occlusal correction, and changing the wires [38]. Teledentistry still has to address the issue regarding the conceptualization of data and information in order to maintain a level of quality equivalent to that of on-site orthodontic consultations [36]. 

### 3.4. In Restorative Dentistry and Endodontics

Endodontics is a highly sophisticated branch of dentistry that is handled by specialist dentists called endodontists. The provision of endodontic care to underprivileged patients is possible through the use of teledentistry. Teledentistry can be used to recognize root canal orifices from a distance, which tends to suggest that highly trained endodontists can help general dentists in the identification of root canal orifices by providing guidance and instruction over the telephone about the recognition of root canals [40]. Teledentistry, which utilizes the internet as a communication medium, has proven to be effective in the diagnosis of periapical lesions of the anterior teeth [41]. It has the dual benefit of lowering the costs associated with long-distance visits and increasing the accessibility of emergency assistance. 

### 3.5. In Pediatric Dentistry 

A child’s overall health and well-being are significantly affected by their oral and dental health [42]. Pediatric dentists can be given the chance to use technological developments in the information and telecommunications field to serve their patients and encourage better oral hygiene habits [43,44,45]. For pediatric patients, preventive counseling can be given over the phone to initiate treatment regimens. Over the telephone or through email, dietary charts can be sent or provided in advance, and introducing patients to online dental hygiene resources is possible. A high-strength sodium fluoride toothpaste prescription might be administered by an online prescription system [46]. Some pediatric patients could be sent to a specialist pediatric dentist for delayed tooth eruption or tooth exfoliation. In this regard, an initial video call for an evaluation of the condition of the oral cavity could be helpful in determining whether or not the patient needs to undergo further professional consideration. In addition, pediatric dentists and orthodontic specialists who wait for the shedding of the deciduous teeth before performing final treatment may find that teledentistry is an advantageous alternative. Other developmental problems such as neonatal or natal teeth and eruption cysts might all be monitored by teledentistry [47]. One of the main benefits of teledentistry is that it eliminates the need for patients and parents to travel and leave their workplace or school in order to see a dentist in person [48]. Nevertheless, the high expense of having a smartphone or computer means that some patients may be unable to use teledentistry.

## 4. Informed Consent between Dentist and Patient

Informed consent is an integral part of the relationship between doctors and patients in every medical field. Teledentistry should cover all aspects of the standard, conventional consent form; in addition, it should also inform the patient of the inherent risk of misdiagnosis and/or treatment due to the involvement of technological failure. Patients should be informed that, despite strong attempts to protect the confidentiality of the patient, their information must be transmitted electronically, and that the data can be intercepted. To prevent malpractice during treatment, the form should include the names of both the referring and consulting doctors, and the consulting doctor should obtain a copy of the consent document prior to establishing any form of patient interaction [16]. Dental practitioners must follow guidelines and procedures for informed consent (verbal or written), patient details, personal communications, and consultancies’ privacy and confidentiality.

## 5. Dentist–Patient Relationships

**Figure 2:**
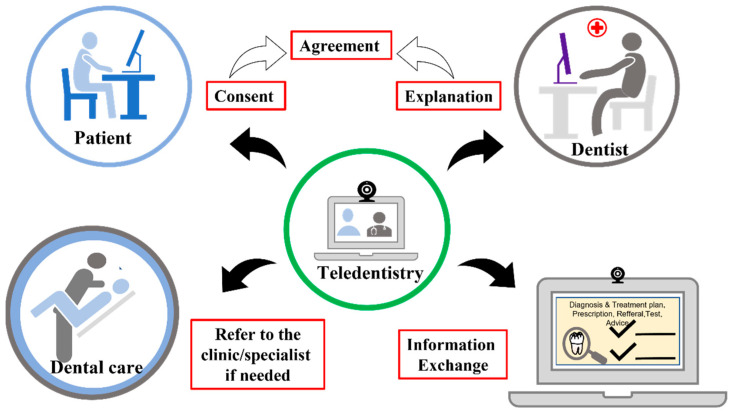
A feasible and practical workflow of an online real-time consultation between patient and dentist.

In the dentist–patient relationship, mutual trust is required when the dentist asks the patient to provide the necessary information and when the patient agrees to the dentist’s treatment policy. Therefore, it is necessary to implement this based on mutual agreement. The agreement should include specific rules for implementing online consultation. However, there is a difference in medical knowledge between dentists and patients. Dentists must provide sufficient information to patients about the advantages of online consultation and the disadvantages that may occur. Therefore, it is necessary to obtain the consent of the patient thoroughly, and then the dentist should make appropriate decisions, including the applicability of online dental care [15]. For this reason, online dental care is used only when there is already a direct relationship between the dentist and the patient. It is required that the same dentist should perform face-to-face online dental care in an appropriate combination (Figure 2).

## 6. Benefits for Patients

In case of emergency, a patient may contact the dentist directly from a remote location. This helps the dentist to evaluate the problem in detail, prior to prescribing any medicine, and thereby can save time, money, and prompt visits to a hospital or clinic. Consequently, there is less need for travel and a shorter wait time in dental offices. Teledentistry is also less costly than face-to-face dental care while also delivering a high-quality service. Furthermore, patients and their families may also select a dentist based on their criteria. This facilitates a second opinion from a dental specialist, who may be geographically far from the patient [49].

## 7. Benefits for Dentists

Since patients do not have to visit the clinic as often, chair times will be reduced. Consultations with dentists could be virtual, and dentists may be able to deal with more patients per day. Patients do not need to attend follow-up visits after receiving treatment because the dentist may simply electronically communicate with the patient. Furthermore, dentists can quickly consult with a specialist if they need a second opinion, with the patient’s consent. Another advantage for dentists is the ability to communicate with patients who live in remote areas [49].

## 8. Responsibility of the Dentists

In teledentistry, the dentists hold the main responsibility for the consultation performed online. For this reason, the dentist carefully judges whether sufficient information is obtained from the online consultation and the information can be used to make an appropriate diagnosis. When using teledentistry for dental consultations, a dentist’s primary responsibility will be to diagnose a disease, develop treatment plans, determine the primary and preventive oral health services that could be provided to patients in their home communities, and expedite referral and follow-up for dental treatment when necessary [50]. In addition, dentists take sufficient information security measures for information communication and the storage of patient information so that the patient’s information will be safe. The dentist should consider every detail of the diagnosis and treatment process for the purpose of treatment, the prognosis of treatment, and further improvement of the diseased condition and complaints that remain unsolved after receiving the consultation, if any. These include (i) patient record acquisition, management, sharing, and storage; (ii) maintenance of the privacy and security of patient health information across the delivery system; (iii) equipment and technology to enable care at multiple endpoints.

## 9. Responsibility of the Patients

Patients might view any licenses or certifications demonstrating that a dentist has been properly trained and approved to provide online teledentistry consultations. Moreover, the patient can ask any questions that come to mind related to their current or previous oral issues. During online consultations, patients may ask questions, report symptoms, and discuss other information, including the ability to review a known problem or condition and upload photos as required [51]. The dentist may wish to obtain additional information from the patient regarding the current oral problem. The patient should cooperate with the dentist by providing additional necessary information when required by the dentist.

## 10. Limitations during Online Consultations

Online consultations have limitations in diagnosing diseases and evaluating patient problems, especially in dentistry. Sometimes, clinicians may be the most significant impediment to implementation, being resistant to change due to various influencing factors, having concerns about the consistency of patient interactions, and being concerned about technological difficulties. The difficulty of assessing the dentition and soft tissue, especially in the posterior oral cavity, would be the utmost limitation of online consultations in this field. Image quality, internet accessibility, and patient considerations including the ability to connect to the software, provide real-time interactive video consultations, proper light arrangement, manual dexterity, and acquaintance with mobile/tablet devices should be taken into consideration. However, the ability to assess extraoral and intraoral swelling, grossly carious teeth, soft tissue lesions, mobile teeth, fractured prosthodontic work, and orthodontic emergencies is highly impractical through online consultation [52]. 

Furthermore, a combination of the store-and-forward approach with real-time video can minimize the limitation of poor image quality. Moreover, clinical examinations are not possible, and video conferencing cannot provide an adequate definition to facilitate oral examination. In addition, special investigations that require direct patient contact and would aid in an accurate diagnosis are difficult to obtain in an online teledentistry consultation [11].

## 11. Dentist–Patient Confidentiality

The dentist–patient relationship is built to understand that any information shared by the patient with the dentist will not be shared without the patient’s consent. Patients have a right to privacy, and they must provide the dentist with comprehensive health records to ensure safe care. Patients should expect to have access to important privacy information, such as who has access to their medical information, with whom their medical information is shared, why any medical information is shared with a third party, how to request copies of their personal information on file, and what medical and personal information is obtained. All of these are common questions that any reputable teledentistry service provider should answer. As a result, a teledentist must follow the guidelines that all doctors must follow regarding patient privacy. Dentists who use teledentistry must take every precaution to safeguard their system and the data that they transmit [51].

## 12. Providing Accurate Information

Online consultation provides less information about the patient’s condition than face-to-face dental care. After correctly understanding the limitations of online consultation by such online dental care, the dentist will explain the advantages of online dental care/treatment and the disadvantages to the patient in advance. Communications with the patient should be appropriately conducted to avoid using technical and complicated terminology [25].

## 13. Teledentistry during COVID-19

**Figure 3:**
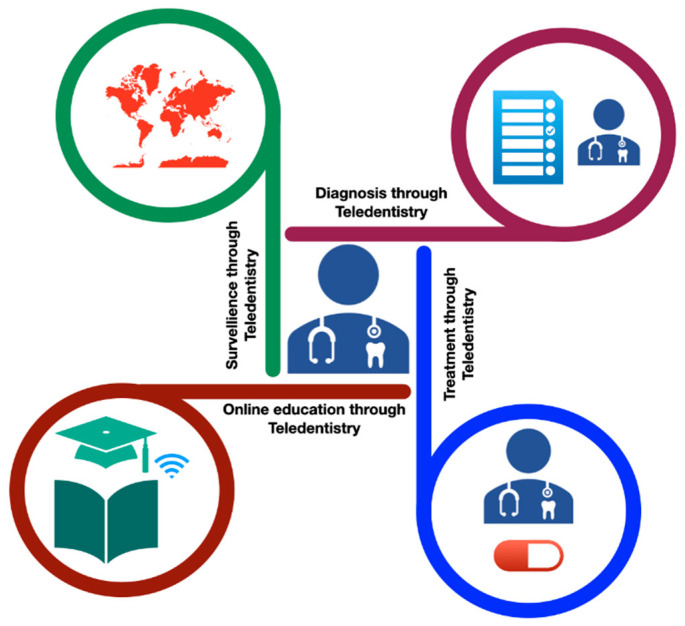
Application of teledentistry during COVID-19 pandemic situation.

Teledentistry is an innovative approach to dentistry and access problems that does not require close contact with the dental professional. Dentistry has been slower than other healthcare areas in terms of communication and information technologies while providing dental care. The rapid adoption of teledentistry by the dental profession during the COVID-19 pandemic offers an excellent opportunity to investigate how teledentistry can be used in dental treatment in the long-term and short-term process, as well as providing a safe and secure environment in which to deliver high-quality care while minimizing the risk of infection. Dentists can use online teledentistry consultation to provide necessary advice, monitor a patient’s condition more than once, and treat patients with emergency dental problems both during and after the COVID-19 pandemic. In the current situation, it will be essential for dental services to have access to a video consultation solution in order to provide a better triage service to those who need it more effectively. Using a video consultation service will aid in limiting patient contact during the COVID-19 outbreak’s recovery and restoration phase, as well as in the future. This will allow practices to manage the backlog of patients currently unable to access dental care and prioritize those with acute therapy problems so that the return of routine dental care can be facilitated over time (Figure 3) [6].

## 14. Acceptance of Teledentistry by Dentist and Patient

Most dentists worldwide may find teledentistry to be a complex system and struggle on many points to adopt these new skills [53,54]. Not only do they find it a technologically challenging procedure, but they are also fearful of making an incorrect diagnosis.

In some cases, the expanded level of cause and expense is also a matter of concern for the dentists. Poor internet connection, a lack of suitable technological devices and support, and inadequate training facilities are the limitations in this field [55]. Furthermore, dentists are confronted by various challenges, such as scarce financial reimbursement, deficient guidelines, the inharmonious relationship between the organization of teledentistry with the healthcare service providers, a lack of coordination, lack of connection between the central and remote centers, and, lastly, the high costs of infrastructure [54]. A lack of ability to perform tooth percussion and pulp vitality tests is an additional constraint.

Accepting the expansion of teledentistry, it is necessary to address the challenges mentioned above. Therefore, dentists should receive sufficient training and develop their knowledge about the technology. Acknowledging the COVID-19 situation, dental healthcare providers should be trained regularly to face the obstacles of transmissible diseases and deal with any pandemic situation by implementing teledentistry. Nevertheless, adequate capitalization, research funds, and validation of teledentistry within the healthcare system should be necessary [55,56].

Acceptance of teledentistry by the patient is fundamental to the success of this component. The patient may perceive that reduced communication with their dentists may occur due to a lack of face-to-face communication, which may lead them to feel anxious about the situation. With the broad expansion of telemedicine, teledentistry will gain more acceptance from patients day by day. Numerous studies have revealed that teledentistry is gradually becoming more recognized by both patients and dentists [56,57]. 

## 15. Recommendations for Effective Online Teledentistry Consultation

An online consultation is the act of examining a patient between a dentist and a patient via an information and communication device and recommending a dental clinic/hospital to see a dentist in real time in teledentistry. Encouragement of consultation with the minimum necessary diagnosis according to the individual patient’s condition, such as selecting the appropriate dental specialized department, is important. Diagnosing specific diseases (if the diagnosis is adequately made), informing patients of the diagnosis, instructing them on the specific use of over-the-counter drugs, prescribing, etc., are all examples of online medical treatment that can also be performed via online teledentistry consultation. In addition, there are cases where follow-up or non-examination instructions are given to persons with symptoms that are clearly not enough to see a dentist or undergo dental treatment according to the patient’s individual condition. General consultation recommendations that do not involve any judgment can be provided as remote health and consultations.

## 16. Future Perspectives of Teledentistry

Although teledentistry has been around since the 1990s, its significance was only recently recognized in the pandemic situation that developed worldwide. Since the pandemic is still ongoing, teledentistry will likely be around for a very long time. Patients who are unable to go outside due to health issues and must remain inside their homes can now obtain dental consultations and treatments with the simple press of a phone button, thanks to the advent of teledentistry. At the same time, elderly patients and those with physical disabilities or who are incarcerated for various reasons can benefit from the emergence of teledentistry, which allows dental care to be delivered remotely. Similarly, for patients living in rural and inaccessible areas, for whom the time to travel to and from the dental clinic is a major setback, teledentistry represents a promising solution. Taking into account that it has such widespread use, as well as the significant number of drawbacks, both for patients and dental specialists, it will only be utilized to an increasing degree in the future. The major innovation in this field is the availability of high-quality software programs that are reasonably priced and simple to use by both patients and professionals. Although its benefits have been addressed, teledentistry cannot be regarded as a substitute for traditional dental care; instead, it can only be considered an addition to conventional dentistry. However, it is safe to state that the intersection of dental and telecommunications is now well established and is expected to progress on a constructive path. This convergence of dentistry and telecommunications is the future of dentistry.

## 17. Conclusions

Currently, advanced medical equipment and instruments have made teledentistry a more convenient way to reach patients on a large scale by providing teleconsultation support at any time and place through internet-based media platforms. Campaigning for public awareness about various health issues and promulgating valuable information can be achieved with an extensive group of targeted patients with the assistance of this media platform, particularly in an emergency. Teledentistry has no alternative to provide a safer patient consultation by lessening the burden of clinics at times of crisis. Hence, teledentistry plays a vital role in serving patients with a dynamic management strategy and fulfills the needs of the patient’s treatment in the best possible way.

## Data Availability

Not applicable.
